# Phosphatidylcholine Extends Lifespan via DAF-16 and Reduces Amyloid-Beta-Induced Toxicity in *Caenorhabditis elegans*

**DOI:** 10.1155/2019/2860642

**Published:** 2019-07-11

**Authors:** So-Hyeon Kim, Bo-Kyoung Kim, Suhyeon Park, Sang-Kyu Park

**Affiliations:** Department of Medical Biotechnology, Soonchunhyang University, Asan 31538, Republic of Korea

## Abstract

Phosphatidylcholine is one of the major phospholipids comprising cellular membrane and is known to have several health-promoting activities, including the improvement of brain function and liver repair. In this paper, we examine the *in vivo* effect of dietary supplementation with phosphatidylcholine on the response to environmental stressors and aging in *C. elegans*. Treatment with phosphatidylcholine significantly increased the survival of worms under oxidative stress conditions. However, there was no significant difference in response to stresses caused by heat shock or ultraviolet irradiation. Oxidative stress is believed to be one of the major causal factors of aging. Then, we examined the effect of phosphatidylcholine on lifespan and age-related physiological changes. Phosphatidylcholine showed a lifespan-extending effect and a reduction in fertility, possibly as a tradeoff for long lifespan. Age-related decline of motility was also significantly delayed by supplementation with phosphatidylcholine. Interestingly, the expressions of well-known longevity-assuring genes, *hsp-16.2* and *sod-3*, were significantly upregulated by dietary intervention with phosphatidylcholine. DAF-16, a transcription factor modulating stress response genes, was accumulated in the nucleus by phosphatidylcholine treatment. Increase of the ROS level with phosphatidylcholine suggests that the antioxidant and lifespan-extending effects are due to the hormetic effect of phosphatidylcholine. Phosphatidylcholine also showed a protective effect against amyloid beta-induced toxicity in Alzheimer's disease model animals. Experiments with long-lived mutants revealed that the lifespan-extending effect of phosphatidylcholine specifically overlapped with that of reduced insulin/IGF-1-like signaling and required DAF-16. These findings showed the antioxidant and antiaging activities of phosphatidylcholine for the first time *in vivo*. Further studies focusing on the identification of underlying cellular mechanisms involved in the antiaging effect will increase the possibility of using phosphatidylcholine for the development of antiaging therapeutics.

## 1. Introduction

Aging is one of the most complex biological processes. During aging, the structure and cellular function of the body gradually decline, while susceptibility to disease and death rapidly increases [1]. To explain the aging process, numerous theories of aging have been suggested. The free radical theory suggests that various free radicals present in the surrounding environment cause cellular damage and accumulation of this damage eventually leads to aging in the organism [2]. Major free radicals are the reactive oxygen species (ROS) produced as a byproduct of mitochondrial electron transport chain reaction. There is a positive correlation between cellular ROS levels and an organism's lifespan [3]. The other related theory of aging is the mitochondrial decline theory of aging, which emphasizes the role of age-related decrease in mitochondrial function in the normal aging process [4]. As cells age, mutations are accumulated in the mitochondrial genome and the efficiency of the mitochondrial electron transport chain reaction declines, producing less ATP and more ROS [5]. Some theories of aging focus on the importance of genomic stability [6]. For example, the telomere theory of aging suggests that the attrition of telomere sequences at chromosome ends as cell replicates play a key role in cellular senescence [6]. Faster attrition of telomere sequences was observed in the genomes of a Werner syndrome patient, which is an adult progeria showing accelerating aging phenotypes [7]. However, there is no single theory of aging that can explain the complex aging process as a whole and people believe that many aging theories are interlinked with each other.

Based on the free radical theory of aging supported by the majority, many genetic and nutritional interventions modulating the cellular antioxidant system have been studied so far. Genetic knockout of antioxidant genes, including catalase (CAT) and superoxide dismutase (SOD), shortened the lifespan of many model organisms, while overexpression of those genes extended the lifespan [8]. However, some studies have reported that additional copy of antioxidant genes had no effect on lifespan [9]. The role of antioxidant genes in lifespan determination is still elusive. Nutritional interventions with antioxidants include dietary supplementation with resveratrol and vitamin E. Resveratrol is a polyphenol compound found in many plants, including grapes, raspberries, cranberries, and other berries. Resveratrol has a variety of beneficial bioactivities, such as antioxidant, anticancer, and anti-inflammatory effects [10]. Supplementation with resveratrol increased lifespan in yeast, *C. elegans*, and *Drosophila melanogaster* [11–13]. The lifespan-extending effect of resveratrol involves the activation of the SIRT1 gene, which inhibits apoptosis [14]. Recent studies have shown that cysteine derivatives have both antioxidant and antiaging effect *in vivo*. N-Acetyl-L-cysteine increased the resistance to environmental stresses and lifespan, mimicking dietary restriction [15]. Supplementation with selenocysteine conferred longevity phenotype and ameliorated age-related pathophysiological changes [16]. Extracts from *Acanthopanax sessiliflorus*, a plant used as a traditional treatment for many diseases, or *Tenebrio molitor*, an insect whose extracts have antibacterial, antifungal, and anticancer activities, also showed antioxidant and antiaging effect in *C. elegans* [17, 18].

Phosphatidylcholine is one of the most abundant phospholipids found in all cell membranes. Recent studies have identified various beneficial health effects of phosphatidylcholine. Impaired biosynthesis of phosphatidylcholine is associated with fatty liver disease and lowered liver regeneration [19]. Phosphatidylcholine also modulates brain function and brain disease. In the aged human, the plasma level of phosphatidylcholine was positively correlated with cognitive flexibility within the prefrontal cortex and the decreased plasma level of phosphatidylcholine was observed in Alzheimer's disease (AD) patients [20]. Dietary supplementation with phosphatidylcholine improved brain function, such as learning and memory, and conferred increased resistance to oxidative stress by modulating the activity of SOD in mice [21]. In rat adrenal pheochromocytoma cells, treatment with phosphatidylcholine hydroperoxides enhanced the activities of antioxidant genes, including CAT, SOD, and glutathione peroxidase [22]. Nanoparticles based on phosphatidylcholine, vitamins, and melatonin showed wrinkle-reducing and antiaging effects in skin [23].

In this study, we investigated the antistress and antiaging effects of phosphatidylcholine in *C. elegans*. We also investigated the effect of phosphatidylcholine on age-related physiological and genetic markers and age-related disease. Finally, we determined the underlying mechanisms involved in the lifespan-extending effect of phosphatidylcholine. This study will broaden the understanding of the aging process itself and provide novel biomolecules having antiaging activity *in vivo*.

## 2. Materials and Method

### 2.1. Worm Strains and Culture Conditions

N2 was used as the wild-type control in all experiments. The long-lived mutants, *age-1* (*hx546*), *clk-1* (*e2519*), and *eat-2* (*ad465*), and the green fluorescent protein- (GFP-) expressing strains, CL2070 (dvIs70 [*Phsp-16.2::GFP, rol-6*]), CF1553 (muIs84 [*Psod-3::GFP, rol-6*]), and TJ356 (zls356 IV [*daf-16p::daf-16a/b::GFP, rol-6*]), were purchased from the *C. elegans* Genetics Center (CGC, Minneapolis/St. Paul, MN, USA). The CL4176 expressing muscle-specific human amyloid beta (A*β*)_1-42_ (dvls27 [*myo-3/Aβ1-42/let UTR, rol-6*]) was used for A*β*-induced toxicity assay. Worms were cultured at 20°C on solid Nematode Growth Medium (NGM) plates (25 mM NaCl, 1.7% agar, 2.5 mg/ml peptone, 50 mM KH2PO4 (pH 6.0), 5 *μ*g/ml cholesterol, 1 mM CaCl_2_, and 1 mM MgSO_4_) spotted with *Escherichia coli* OP50 as food source.

### 2.2. Resistance to Oxidative Stress

Five young adult worms were transferred to a fresh NGM plate and permitted to lay eggs for 6 h. Then, the five adult worms were removed from the plate. The remaining eggs were hatched and grown on NGM plates for 3 days at 20°C. Thirty age-synchronized worms were transferred to fresh NGM plates containing different concentrations (1, 10, and 100 mg/l) of phosphatidylcholine and adapted for 24 h in 20°C. Then, worms were placed in 96-well plates (5 worms/well) containing 2 mM hydrogen peroxide (H_2_O_2_) in S-basal without cholesterol (5.85 g sodium chloride, 1 g potassium phosphate dibasic, and 6 g potassium phosphate monobasic for 1 l sterilized distilled water). The survival of worms was recorded. A worm not responding to any mechanical stimuli was considered as dead. For statistical analysis, the log-rank test was used [24].

### 2.3. Thermotolerance Assay

Sixty age-synchronized worms were transferred to fresh NGM plates pretreated with different concentrations of phosphatidylcholine (1, 10, and 100 mg/l) and incubated at 20°C for 24 h. Then, worms were exposed to 35°C heat shock for 7 h. After heat shock, worms were transferred back to a 20°C incubator. On the next day, the survival of worms was monitored every day, until all worms were dead.

### 2.4. Survival after Ultraviolet (UV) Irradiation

Sixty age-synchronized young adult worms were transferred to fresh NGM plates containing different concentrations of phosphatidylcholine (1, 10, and 100 mg/l). After 24 h at 20°C, worms were irradiated with 20 J/cm^2^/min of UV for 1 min in a UV crosslinker (BLX-254, Vilber Lourmat Co., Torcy, France). Then, worms were transferred to fresh NGM plates treated with different concentrations of phosphatidylcholine. Living and dead worms were scored daily, until all worms were dead.

### 2.5. Lifespan Assay

To prevent internal hatching during the assay, 5-fluoro-2′-deoxyruridine (12.5 mg/l) was added to NGM plates. With sixty age-synchronized worms, the numbers of live and dead worms were recorded every day. Worms lost, killed, or having internal hatching were excluded from the assay. The log-rank test was employed for statistical comparison of survival curves [24]. A *P* value lower than 0.05 was considered to be a significant difference between two survival curves.

### 2.6. Fertility Assay

Five L4/young adult stage worms were transferred to a fresh NGM plate containing different concentrations of phosphatidylcholine (10 and 100 mg/l) and permitted to lay eggs for 5 h. The eggs were maintained at 20°C for 2 d. Ten 2-day-old worms were transferred to 10 fresh NGM plates individually containing different concentrations of phosphatidylcholine every day. Eggs spawned each day by an individual worm were incubated at 20°C for 48 h, and the number of progeny produced was recorded, until the worm no longer produced eggs.

### 2.7. Motility Assay

Age-synchronized young adult worms were grown on NGM plates containing different concentrations of phosphatidylcholine (10 and 100 mg/l) at 20°C. On 5, 10, 15, 20, and 25 days after laying eggs, worms were classified according to their motility: phase 1, a worm moving without any mechanical stimulation, phase 2, a worm that moves in response to mechanical stimuli, and phase 3, a worm that can move only the head part with a mechanical stimulus. The relative distributions of each phase and dead worms among 100 age-synchronized worms were compared between the untreated control and phosphatidylcholine-treated groups. For quantitative analysis, thrashing assay was performed. After treating phosphatidylcholine to age-synchronized worms, fifteen worms were randomly selected and placed on NGM plates individually for 2 min. Then, a single worm was transferred to M9 buffer and adapted for 1 min. The number of trashing per 1 min was counted for each worm.

### 2.8. Subcellular Localization of DAF-16

Sixty age-synchronized TJ356 worms were transferred to NGM plates with or without 100 mg/l of phosphatidylcholine. After 5, 7, and 9 days, worms were anesthetized with 1 M sodium azide on a slide glass and the cellular distribution of DAF-16 was monitored using a fluorescence microscope.

### 2.9. Expression of Longevity Assurance Genes

Age-synchronized CL2070 and CF1553 worms (*n* = 20) were grown on NGM plates containing 10 or 100 mg/l of phosphatidylcholine for 5, 7, and 9 days. Then, a single worm was mounted on a slide glass coated with 2% agarose, anesthetized with 1 M sodium azide, and covered with cover slide glass and the expression level of GFP was monitored with a confocal microscope (Olympus FV10i, Olympus, Tokyo, Japan). The quantification of GFP expression was determined with a fluorescence multireader (Infinite F200, Tecan, Grodig, Austria).

### 2.10. Cellular ROS Levels

Age-synchronized young-adult worms were treated with or without phosphatidylcholine for 5 and 7 days at 20°C. Then, worms were transferred to a 96-well black plate containing 190 *μ*l of PBST individually (*n* = 20). Incubate worms for 3 h with 10 *μ*l of H2DCF-DA (Sigma-Aldrich, St. Louis, USA), and fluorescence intensity was measured with a fluorescence multireader (Infinite F200, Tecan, Grodig, Austria).

### 2.11. A*β*-Induced Toxicity Assay

Thirty young adult CL4176 worms grown at 15°C were transferred to NGM plates pretreated with 10 or 100 mg/l of phosphatidylcholine and permitted to lay eggs for 2 h at 15°C. Then, all adult worms were removed from the plate and the progeny were grown for 24 h at 15°C. Then, sixty randomly selected worms were incubated in 25°C incubator for 24 h to induce human A*β* expression. The number of paralyzed worms was counted every hour.

### 2.12. RNA Interference (RNAi)

For the gene knockdown of *daf-16*, *E. coli* clones harboring *daf-16* gene for RNAi were obtained from the Ahringer RNAi library [25]. The expression of double-stranded RNA was induced by 0.4 mM isopropyl-*β*-D-thiogalactoside (IPTG) (Sigma-Aldrich, St. Louis, MO, USA) for 4 h after OD600 reached 0.4. Then, cultured bacteria were used as food source for RNAi experiment. *E. coli* clone transformed with empty vector was used as a negative control for RNAi.

## 3. Results

### 3.1. Phosphatidylcholine Increased Resistance to Oxidative Stress and Lifespan

**Figure 1:**
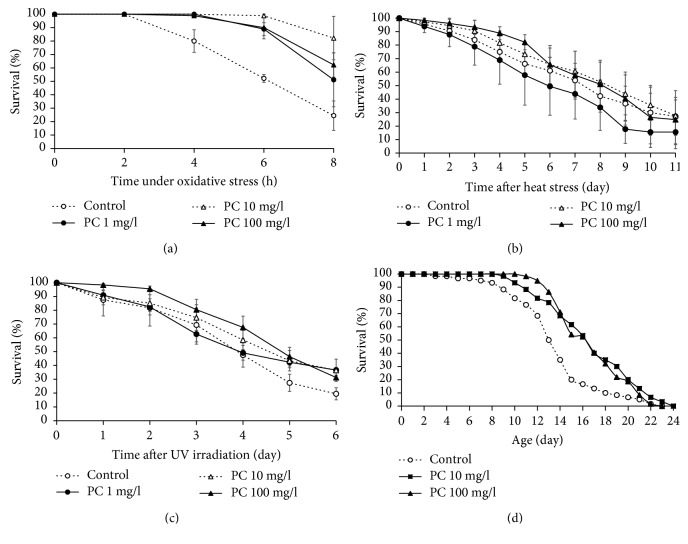
Effect of phosphatidylcholine on resistance to oxidative stress and lifespan. Sixty age-synchronized worms pretreated with phosphatidylcholine were placed under (a) oxidative stress, (b) heat shock, and (c) UV irradiation conditions. Survival of worms was monitored at indicated times after stress. (d) Lifespan was compared between the untreated control and worms treated with phosphatidylcholine. Error bar indicates standard error. PC: phosphatidylcholine.

In order to investigate the effect of phosphatidylcholine on the response to environmental stresses, we examined the effect of phosphatidylcholine on resistance to oxidative stress, heat shock, and UV irradiation. H_2_O_2_ was used to induce oxidative stress in *C. elegans*. A significant increase in survival under oxidative stress conditions was observed in worms supplemented with phosphatidylcholine (Figure 1(a)). The mean survival time was 3.6 h in the untreated control. Pretreatment of phosphatidylcholine increased the mean survival time under oxidative stress condition up to 6.3 (*P* = 0.256), 7.0 (*P* = 0.027), and 6.9 h (*P* = 0.032) with 1, 10, and 10 mg/l of concentration. However, lower concentration of phosphatidylcholine than 1 mg/l failed to show a significant change in resistance to oxidative stress (data not shown). Then, we determined the effect of dietary supplementation with phosphatidylcholine on other environmental stresses. However, unlike the results obtained with oxidative stress, there was no significant difference in resistance to either heat shock or UV irradiation. The survival curve after 7 h of heat shock was not altered by any concentration of phosphatidylcholine tested (Figure 1(b)). Dietary supplementation with phosphatidylcholine failed to increase the time course survival rate after UV irradiation (Figure 1(c)). Taken together, we concluded that phosphatidylcholine positively regulates resistance to oxidative stress but has no effect on the response to heat shock or UV irradiation. The free radical theory of aging suggests that the age-related accumulation of cellular damages caused by oxidative stress is one of the major causal factors of aging [2, 3]. Based on the previous finding that phosphatidylcholine can increase the resistance to oxidative stress, we asked whether dietary supplementation with phosphatidylcholine can modulate lifespan in *C. elegans*. As shown in Figure 1(d), the lifespan of *C. elegans* was significantly increased by phosphatidylcholine. The mean lifespan of wild-type N2 was 13.8 days. Animals treated with 10 mg/l of phosphatidylcholine showed a 28.8% increase in mean lifespan (17.7 d, *P* < 0.001). There was also a significant increase of mean lifespan in 100 mg/l of phosphatidylcholine-treated worms: the mean lifespan was 16.8 days (22.2% increase, *P* < 0.001) (Figure 1(d)). Independent repetitive experiments also showed a significant increase in lifespan by supplementation with phosphatidylcholine (Table 1). To discern whether this effect of phosphatidylcholine is caused directly by phosphatidylcholine or indirectly by bacteria with phosphatidylcholine treatment, we performed the lifespan assay with dead bacteria. The same significant lifespan-extending effect was observed by phosphatidylcholine in worms fed with dead bacteria, suggesting that the longevity phenotype was induced directly from phosphatidylcholine (Figure S1).

### 3.2. Fertility Was Reduced by Supplementation with Phosphatidylcholine

**Figure 2:**
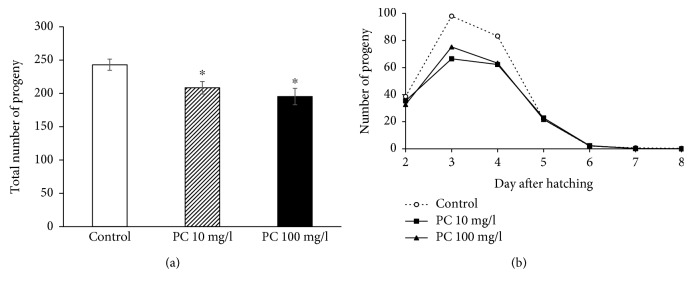
Effect of phosphatidylcholine on reproduction. (a) Total number of progeny produced during a gravid period was compared between the untreated control and phosphatidylcholine-treated groups. (b) Time course distribution of progeny produced during a gravid period. Number of progeny was recorded every day until there was no progeny produced. Data indicate a mean of 10 individual worms. Error bar indicates standard error. PC: phosphatidylcholine; ^∗^statistically significant (*P* < 0.05).

Many lifespan-extending genetic/dietary interventions have shown reduced fertility as a tradeoff [12, 26]. We examined the effect of phosphatidylcholine on the reproduction of *C. elegans*. The total number of progeny produced during a gravid period significantly decreased by supplementation with phosphatidylcholine (Figure 2(a)). In wild-type N2 worms, 243.0 ± 8.41 progeny were produced. However, the number of total progeny was reduced to 208.4 ± 9.43 (*P* = 0.012) in the 10 mg/l phosphatidylcholine-treated group and 195.3 ± 12.25 (*P* = 0.005) in the 100 mg/l phosphatidylcholine-treated group. The time course distribution of progeny produced during a gravid period revealed that there were decreases in the number of progeny on the 3rd day and 4th day after laying eggs (Figure 2(b)). Independent replicative experiment also showed the same reduced fertility by dietary intervention with phosphatidylcholine (data not shown). We also examined effect of phosphatidylcholine on fertility in *age-1* mutants, which is known to have reduced fertility as cost for extended lifespan [26]. Interestingly, there was no significant change in fertility by supplementation with phosphatidylcholine in *age-1* mutants (Figure S2). Our results indicate that the longevity phenotype conferred by supplementation with phosphatidylcholine accompanies reduced fertility as a tradeoff for long lifespan.

### 3.3. Age-Related Decline in Motility Was Delayed by Phosphatidylcholine

**Figure 3:**
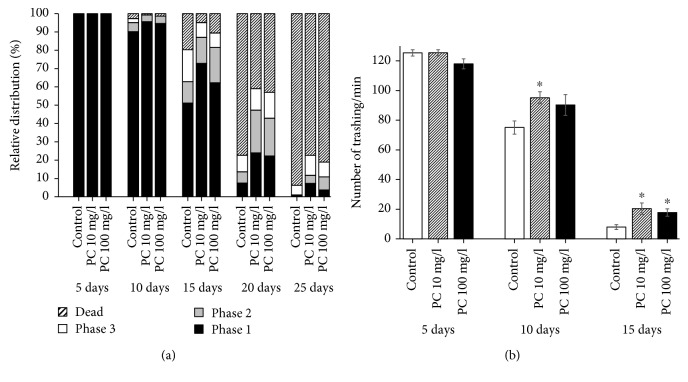
Effect of phosphatidylcholine on age-related decline in motility. (a) Relative distribution of worms in different locomotive phases was calculated in the untreated control and phosphatidylcholine-treated groups at indicated days. Phase 1, worms moving spontaneously without any stimuli; Phase 2, worms moving the whole body in response to mechanical stimuli; Phase 3, worms moving only the head part in response to mechanical stimuli; (b) the number of trashing was counted individually (*n* = 15) at indicated days after laying eggs. PC: phosphatidylcholine; ^∗^statistically significant (*P* < 0.05).

One of the obvious physiological changes happening with aging in almost all organisms is muscle atrophy and reduced motility [27]. In *C. elegans*, locomotive behavior declines with aging. Then, we investigated the role of dietary intervention with phosphatidylcholine on the age-related decline of motility in *C. elegans*. We could observe the delayed decline of locomotive activity with aging in worms treated with phosphatidylcholine (Figure 3(a)). There were no clear differences in locomotive activity between the untreated control and phosphatidylcholine-treated groups in young worms (5- and 10-day-old worms). In 15-day-old worms, we could detect a slight increase in the number of worms categorized as phases 1 and 2, which are worms moving spontaneously without any mechanical stimuli and worms moving after mechanical stimuli, respectively. In contrast, more worms were classified as phase 3 (worms could move only the head part after mechanical stimuli) in the untreated control, compared to the phosphatidylcholine-treated groups. These differences were not statistically significant (*P* > 0.05). However, a significant difference between the untreated control and phosphatidylcholine-treated groups was detected in 20- and 25-day-old worms. On the 20th day, the number of worms categorized as phase 1 increased from 7.5 ± 1.13% in the untreated control to 24.0 ± 3.46% (*P* = 0.011) with 10 mg/l phosphatidylcholine treatment and 22.3 ± 4.91% (*P* = 0.043) with 100 mg/l phosphatidylcholine treatment. The worms classified as phase 2 also significantly increased by supplementation with phosphatidylcholine: 6.1 ± 0.55, 23.3 ± 3.84 (*P* = 0.011), and 20.6 ± 3.71% (*P* = 0.018) in the untreated control, 10 mg/l phosphatidylcholine-treated group, and 100 mg/l phosphatidylcholine-treated group, respectively (Figure 3(a)). The same significant delay of decline in motility was observed in 25-day-old worms (Table 2). We also examined effect of phosphatidylcholine on thrashing activity. There was no significant difference in the number of thrashing between the untreated control and phosphatidylcholine-treated groups in 5-day-old young animals. However, in aged worms, the number of thrashing was significantly increased by supplementation with phosphatidylcholine (Figure 3(b)). The number of thrashing per min was increased from 75.1 ± 4.45 in the untreated control to 95.1 ± 4.05 (*P* = 0.002) and 90.3 ± 7.06 (*P* = 0.079) in 10 mg/l and 100 mg/l phosphatidylcholine-treated groups, respectively, on 10 days after laying eggs. In 15-day-old control worms, the number of thrashing per min was decreased to 7.9 ± 1.66. However, supplementation with phosphatidylcholine significantly enhanced thrashing activity. The numbers of thrashing per min were 20.3 ± 3.86 (*P* = 0.016) with 10 mg/l of phosphatidylcholine and 17.7 ± 2.50 (*P* = 0.008) with 100 mg/l of phosphatidylcholine (Figure 3(b)).

### 3.4. Phosphatidylcholine Induced Nuclear Localization of DAF-16 and Expression of Longevity Assurance Genes

**Figure 4:**
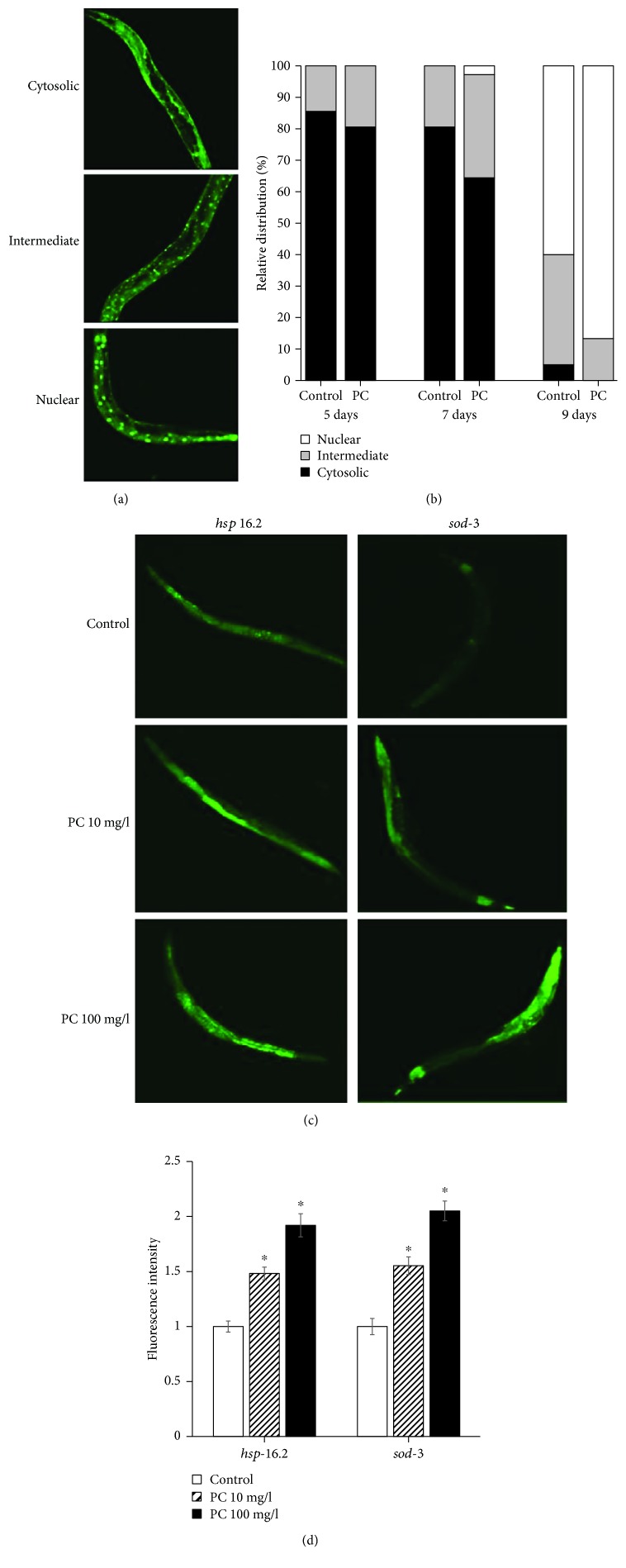
Cellular distribution of DAF-16 and GFP expressions of downstream targets of DAF-16. (a) Subcellular localization was classified as three categories: cytosolic, fluorescence was spread in cytosol; intermediate, GFP can be found both in cytosol and nucleus; and nucleus, clear localization of GFP into the nucleus. (b) Relative distribution of DAF-16 was compared between the untreated control and 100 mg/l phosphatidylcholine-treated groups. (c) Age-synchronized 3-day-old worms were treated with each concentration of phosphatidylcholine for 7 d. Then, worms were observed on confocal microscopy. (d) Change in the expression level was determined using a fluorescence multireader. Fluorescence intensity of PC-treated worms is expressed as the ratio of fluorescence intensity determined in the untreated control. Error bar indicates standard error. PC: phosphatidylcholine; ^∗^statistically significant (*P* < 0.05).

DAF-16 localizes to the nucleus in response to various stresses and modulates the expression of stress response genes [28]. Here, we determined the subcellular distribution of DAF-16 with or without dietary supplementation with phosphatidylcholine (Figure 4(a)). As shown in Figure 4(b), supplementation with phosphatidylcholine induced rapid nuclear localization of DAF-16. In 7-day-old worms, the percentage of worms showing intermediate localization were 19.4 ± 10.56 in the untreated control group and 32.8 ± 11.07 in the phosphatidylcholine-treated group. In the phosphatidylcholine-treated group, 2.8 ± 2.78% of worms showed nuclear localization, while no worm showed nuclear localization of DAF-16 in the untreated control. The differences observed on day 7 were not statistically significant (*P* > 0.05). However, there were significant differences in the subcellular distribution of DAF-16 between the untreated control and the phosphatidylcholine-treated groups on day 9. No worm showed cytosolic distribution of DAF-16 by supplementation with phosphatidylcholine, but 5.0 ± 2.55% of the untreated worms still showed cytosolic distribution of DAF-16. The percent of worms showing intermediate distribution decreased from 35.0 ± 2.55% in the untreated control to 13.3 ± 5.09% in the phosphatidylcholine-treated group (*P* = 0.019). In contrast, more worms showed nuclear localization by supplementation with phosphatidylcholine: 60.0 ± 5.00% in the untreated control and 86.7 ± 5.09% in the phosphatidylcholine-treated group (*P* = 0.020) (Figure 4(b)). Previous studies have shown that the expressions of downstream targets of DAF-16, *hsp-16.2* and *sod-3*, were positively correlated with the individual's lifespan in *C. elegans* [29, 30]. Having observed increased nuclear localization of DAF-16 by phosphatidylcholine, we next analyzed the expression of longevity assurance genes, *hsp-16.2* and *sod-3*, quantitatively. As shown in Figure 4(c), we could detect brighter fluorescence derived by *hsp-16.2* in worms treated with phosphatidylcholine. Quantification of fluorescence using multireader revealed that there was a significant increase in phosphatidylcholine-treated worms compared to the untreated control (Figure 4(d)). Relative expressions were 100.0 ± 5.01 in the 7-day-old untreated control, 148.2 ± 5.74 with 10 mg/l of phosphatidylcholine (*P* < 0.001), and 192.0 ± 10.49 with 100 mg/l of phosphatidylcholine (*P* < 0.001). The expression of *sod-3* was also significantly upregulated by supplementation with phosphatidylcholine (Figure 4(c)). There was a 55.3 ± 7.99% increase in relative expression with 10 mg/l of phosphatidylcholine (*P* < 0.001) and 105.1 ± 8.97% increase with 100 mg/l of phosphatidylcholine (*P* < 0.001) in 7-day-old worms (Figure 4(d)). We could also observe a significant induction of *hsp-16.2* and *sod-3* by supplementation with phosphatidylcholine in 5- and 9-day-old worms (data not shown). This suggests that dietary supplementation with phosphatidylcholine may extend the lifespan of *C. elegans* through an induction of longevity assurance genes.

### 3.5. The Cellular ROS Level Was Increased by Supplementation with Phosphatidylcholine

**Figure 5:**
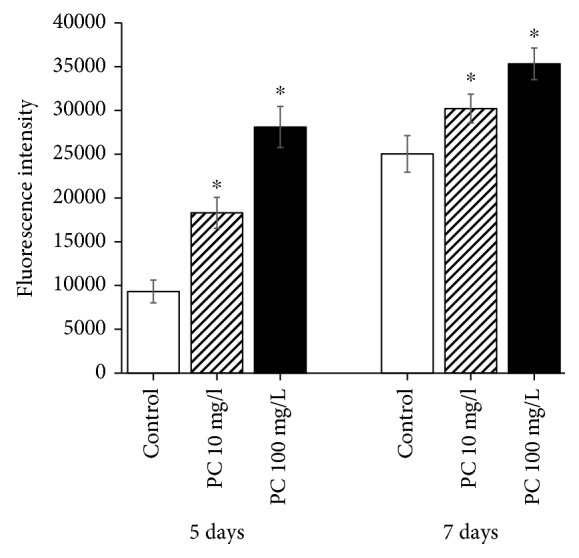
Effect of phosphatidylcholine on the cellular ROS level. The cellular ROS level was measured in an individual worm at indicated days after laying eggs. Error bar indicates standard error. PC: phosphatidylcholine; ^∗^statistically significant (*P* < 0.05).

Having observed increased resistance to oxidative stress and induction of oxidative stress response genes, we, next, tested the effect of phosphatidylcholine on the cellular ROS level. Surprisingly, the cellular ROS level was rather increased by supplementation with phosphatidylcholine (Figure 5). In 5-day-old worms, fluorescence intensity observed in the untreated control was 9322.4 ± 1298.85, which was increased up to 18295.7 ± 1770.11 with 10 mg/l of phosphatidylcholine (*P* < 0.001) and 28105.3 ± 2342.10 with 100 mg/l of phosphatidylcholine (*P* < 0.001). There was an increase in cellular ROS levels in 7-day-old worms, compared to 5-day-old worms in all experimental groups. We could observe the similar increase in the ROS level with phosphatidylcholine in 7-day-old worms. Fluorescence intensities were 25033.1 ± 2092.52, 30217.5 ± 1643.30 (*P* = 0.059), and 35329.4 ± 1806.83 (*P* < 0.001) in the untreated control, 10 mg/l phosphatidylcholine-treated, and 100 mg/l phosphatidylcholine-treated groups, respectively.

### 3.6. Phosphatidylcholine Alleviated A*β*-Induced Toxicity, Which Is Independent of DAF-16

**Figure 6:**
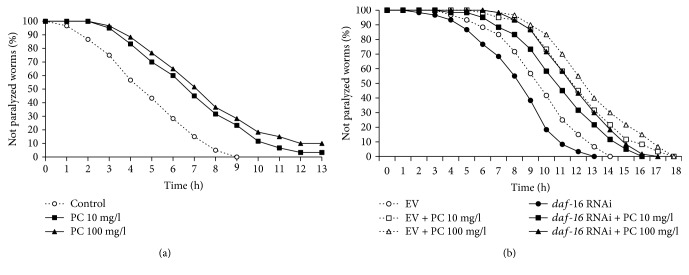
Effect of phosphatidylcholine on A*β*-induced toxicity. (a) Paralyzed worms were counted every hour after human A*β* induction in muscle tissues. (b) Effect of *daf-16* knockdown on reduced susceptibility to A*β*-induced toxicity was determined using RNAi. PC: phosphatidylcholine; EV: empty vector.

Next, we examined the effect of phosphatidylcholine on AD, the age-related neurodegenerative disease. Using the *C. elegans* genetic model of AD, in which human A*β* transgene can be induced in muscle tissues, we determined the rate of paralysis caused by the accumulation of A*β* in muscle [31]. The rate of paralysis was significantly reduced by dietary supplementation with phosphatidylcholine (Figure 6(a)). In the untreated control, the time when 50% of worms were paralyzed was 4.1 h. However, treatment with phosphatidylcholine extended the time when 50% of worms were paralyzed up to 6.3 h with 10 mg/l phosphatidylcholine (*P* < 0.001) and 7.0 h with 100 mg/l phosphatidylcholine (*P* < 0.001). The protective effect by phosphatidylcholine against A*β*-induced paralysis was 55.7 and 71.3% with 10 and 100 mg/l of phosphatidylcholine, respectively. Independent replicative experiments also showed the significant protective effect of phosphatidylcholine on A*β*-induced toxicity (Table 3). It was reported that DAF-16, the FOXO transcription factor involved in insulin/IGF-1-like signaling, can delay the onset of A*β*-induced toxicity [32]. However, we observed the same significant delayed paralysis by supplementation with phosphatidylcholine with *daf-16* knockdown genetic background (Figure 6(b)). These findings suggest that phosphatidylcholine has a protective effect against A*β*-induced toxicity, which is independent of DAF-16.

### 3.7. Effect of Phosphatidylcholine on Lifespan Specifically Overlapped with That of Age-1 Mutation and Requires DAF-16

**Figure 7:**
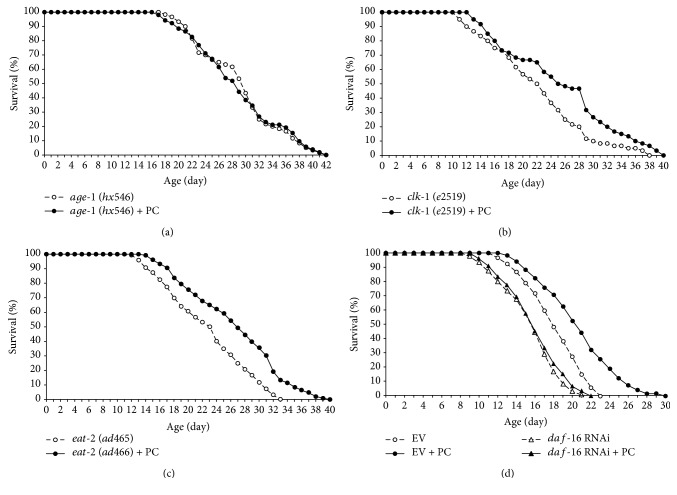
The underlying mechanism involved in the lifespan-extending effect of phosphatidylcholine. Survival curve was compared between the untreated control and phosphatidylcholine-treated groups in three long-lived mutants, (a) *age-1*, (b) *clk-1*, and (c) *eat-2*. (d) Requirement of DAF-16 on lifespan extension by phosphatidylcholine. EV: empty vector; PC: 100 mg/l of phosphatidylcholine.

In order to identify the underlying mechanisms involved in phosphatidylcholine-induced longevity, we tested the effect of phosphatidylcholine on the lifespan of long-lived mutants. The lifespan of *age-1*, in which lifespan was extended due to reduced insulin/IGF-1-like signaling, was not altered by phosphatidylcholine treatment (Figure 7(a)). Interestingly, supplementation with phosphatidylcholine significantly increased the lifespan of *clk-1* and *eat-2*. The *clk-1* (*e2519*) mutant has defect in the ubiquinone biosynthesis required for the mitochondrial electron transport system and, as a result, produces less ROS [33]. The *eat-2* (*ad465*) mutation causes a reduced food pumping rate and leads to dietary restriction as a consequence [34]. There was a 14.7% increase in the mean lifespan of *clk-1* (*e2519*) by supplementation with phosphatidylcholine: 22.2 days in the untreated control and 25.4 days in the phosphatidylcholine-treated group (*P* = 0.013) (Figure 7(b)). The long lifespan of the genetic model of dietary restriction, *eat-2* (ad465), was further extended by phosphatidylcholine treatment. The mean lifespan was increased from 21.0 to 25.8 days by phosphatidylcholine (*P* = 0.001, 18.3% increase) (Figure 7(c)). A repetitive experiment showed the same effect of phosphatidylcholine on long-lived mutants (Table 4). Overall, our data indicate that the lifespan-extending effect of phosphatidylcholine overlaps with that of *age-1* mutation, but not with that of *clk-1* or *eat-2* mutation. The longevity phenotype conferred by reduced insulin/IGF-1-like signaling requires DAF-16 [26]. Based on our finding that the effect of phosphatidylcholine on lifespan overlapped with that of *age-1* mutation, we examined the effect of *daf-16* knockdown on the lifespan extension induced by supplementation with phosphatidylcholine. Unlike the results observed in worms treated with empty vector, dietary supplementation with phosphatidylcholine failed to increase lifespan when the expression of *daf-16* was inhibited using RNAi (Figure 7(d)). Mean lifespan was increased from 17.9 to 21.1 days by supplementation with phosphatidylcholine in worms treated with empty vector RNAi (*P* < 0.001). In contrast, there was no significant difference between the control and the phosphatidylcholine-treated groups in worms treated with *daf-16* RNAi: mean lifespans were 15.5 and 15.6 days in the control and phosphatidylcholine-treated groups, respectively (*P* = 0.622). A replicative experiment showed the same results (Table 5). These results indicate that DAF-16 is required for the effect of phosphatidylcholine on lifespan and support our previous finding that the longevity phenotype conferred by supplementation with phosphatidylcholine is mediated by reduced insulin/IGF-1-like signaling.

## 4. Discussion

Based on the free radical theory of aging emphasizing the role of oxidative damages accumulated with time in normal aging, numerous studies have reported the effect of supplementation with antioxidant on aging. Resveratrol, a polyphenol compound rich in red wine, has been shown to have strong antioxidant and lifespan-extending effects on various model organisms [35]. Recent studies reported that amino acid derivatives, including N-acetyl-L-cysteine, S-allylcysteine, and selenocysteine, increase resistance to oxidative stress and extend lifespan in *C. elegans* [15, 16, 36]. In the present study, we showed that phosphatidylcholine, a phospholipid composing cellular membrane, had an antioxidant activity *in vivo* and conferred the longevity phenotype in *C. elegans* for the first time. Our findings support the free radical theory of aging and provide a scientific background for the use of phospholipid as a novel antioxidant and antiaging biomolecule. There is another well-known theory of aging, named “the membrane theory of aging” by Dr. ImreZs-Nagy [37]. According to the membrane theory of aging, age-related decline in membrane function leads to inefficient communication through membrane and accumulation of toxic compound in the cellular membrane and eventually causes the aging of cells [37]. The amount of phosphatidylcholine in membrane decreases with aging, which results in decreased membrane function for nutrient uptake and toxin excretion and solidification of membrane filled with cholesterol and toxic deposits, called lipofuscin [38]. Since we observed the positive effect of phosphatidylcholine on lifespan, it is also suggestive that supplementation with phosphatidylcholine might reverse the aging process, possibly through the avoidance of age-related depletion of phosphatidylcholine and maintenance of membrane integrity.

The disposable soma theory states that limited cellular resources should be allocated to cell maintenance, repair, and reproduction and that there is a tradeoff between increased lifespan and reduced fertility [39]. We observed a significant decrease in the number of progeny produced in worms treated with phosphatidylcholine, compared to the untreated control, which supports the disposable soma theory of aging. Long-lived *age-1* mutants showed reduced fertility, and knockout of germ cells increased lifespan in *C. elegans* [26]. Lifespan extension by dietary interventions with resveratrol also accompanied decreased reproduction [12]. The other widely used phenotypic marker of aging is the age-related decline of motility. Decreased motility with aging is associated with muscle atrophy and dysfunction [40]. Recent studies have shown that genetic intervention with antioxidant genes, such as *cat* and *sod-1*, or nutritional intervention with antioxidants, such as silymarin and selenocysteine, can modulate age-related muscle dysfunction [16, 41, 42]. Here, we showed that phosphatidylcholine also has a preventive effect against the age-related decline of motility. Since muscle tissues are one of the high energy-demanding tissues and have many mitochondria producing ROS as a byproduct of ATP generation, the effect of phosphatidylcholine on muscle aging seems to be due to its antioxidant activity. In addition to phenotypic age-related markers, we also examined the effect of phosphatidylcholine on the genetic markers of aging. Rea *et al.* found that the variability observed in lifespan among animals with the same genetic and environmental backgrounds was due to the differential expression of *hsp-16.2* [29]. Another study reported that *sod-3* could be a transcriptional marker of long lifespan [30]. The expressions of both *hsp-16.2* and *sod-3* were significantly upregulated by supplementation with phosphatidylcholine. Additionally, we observed increased nuclear localization of DAF-16, a transcription factor-regulating expression of many stress-responsive genes, including *hsp-16.2* and *sod-3* [28]. Taken together, we concluded that dietary supplementation with phosphatidylcholine can modulate the physiological and molecular markers of aging, as well as the organism's lifespan. Since there was an increase in the cellular ROS level with supplementation with phosphatidylcholine, it is suggestive that phosphatidylcholine may be a ROS generator *in vivo* and the antioxidant and antiaging effects of phosphatidylcholine may be due to its hormetic effect. A previous study also showed that a ROS generator, juglone, induced expressions of *hsp-16.2* and hormesis [43].

AD is a neurodegenerative disease whose incidence is associated with aging. One of the molecular markers positively correlated with the incidence of AD is A*β* accumulation in the brain [44]. The Amyloid precursor protein (APP) is degraded by *α*-secretase and produces nonamyloidogenic fragment, but proteolysis of APP by *γ*-secretase produces A*β*. The accumulation of A*β* is found in the brain of AD patients [45]. Lifespan-extending amino acid derivatives, N-acetyl-L-cysteine and selenocysteine, reduced A*β*-induced toxicity in *C. elegans* [15, 16]. DAF-16 is necessary for protection against A*β*-induced toxicity and selenocysteine induced nuclear localization of DAF-16 [16, 32]. We observed that dietary supplementation with phosphatidylcholine can delay paralysis caused by A*β* induction and the effect was not affected by DAF-16. These findings suggest that phosphatidylcholine can modulate A*β*-induced toxicity, which is independent of DAF-16, and can be a strong candidate for the development of functional food for the treatment of AD.

Genetic screenings have identified several lifespan-extending mechanisms in *C. elegans*. The first long-lived mutant reported was *age-1* mutant [46]. The *age-1* gene encodes phosphoinositide 3-kinase, which mediates insulin/IGF-1-like signaling. Mutations in *daf-2*, the upstream receptor gene for the insulin/IGF-1-like signaling pathway, also resulted in increased lifespan [47]. The longevity phenotype conferred by reduced insulin/IGF-1-like signaling is common in various organisms [48]. The other lifespan-extending mutations found in *C. elegans* include mutations in genes causing lowered mitochondrial electron transport chain reaction and decreased production of harmful ROS as a result. For example, mutations in *clk-1* (gene required for the biosynthesis of ubiquinone) or *isp-1* (subunit of mitochondrial complex III) significantly increased lifespan and genome-wide RNAi screening revealed mutations in many genes involved in mitochondrial electron transport chain reaction that led to lifespan extension [33, 49]. The only intervention that showed consistent lifespan-extending effect on all experimental organisms from yeast to monkeys is dietary restriction [50]. The *eat-2* mutant is a well-known genetic model of dietary restriction in *C. elegans* [51]. We tested the effect of supplementation with phosphatidylcholine on the lifespan of *age-1*, *clk-1*, and *eat-2* mutants. Interestingly, only the lifespan of *age-1* was not affected by supplementation with phosphatidylcholine. In addition, phosphatidylcholine did not affect the fertility of *age-1*, while it significantly reduced the fertility of wild-type control. These results indicate that the lifespan-extending mechanism regulated by supplementation with phosphatidylcholine overlaps specifically with reduced insulin/IGF-1-like signaling. The complete disappearance of the longevity phenotype conferred by supplementation with phosphatidylcholine by RNAi knockdown of *daf-16*, the downstream effector of reduced insulin/IGF-1-like signaling, further supports our conclusion.

## 5. Conclusions

In the present study, we report the antioxidant and antiaging activities of phosphatidylcholine for the first time. Phosphatidylcholine also showed a positive effect on the physiological and molecular markers of aging and a protective effect against A*β*-induced toxicity. Finally, we identify the cellular mechanisms underlying the lifespan-extending effect of phosphatidylcholine. Our data reveal the novel bioactivities of phosphatidylcholine and provide a scientific rationale for additional aging research with other phospholipid molecules. The results of the study can also be useful for the development of pharmaceutical or dietary supplement having antiaging effect. Further studies focusing on the antiaging effect of phosphatidylcholine on higher model organisms, such as mice, should follow in the near future.

## Figures and Tables

**Table 1:** Effect of phosphatidylcholine on lifespan of *C. elegans.*

	PC (mg/l)	Mean lifespan (day)	*P* value^1^	% effect^2^
1st experiment	0	13.8		
	10	17.7	<0.001	28.8
	100	16.8	<0.001	22.2
2nd experiment	0	18.6		
	10	21.8	<0.001	17.3
	100	20.3	0.021	9.2
3rd experiment	0	18.7		
	10	20.1	0.078	7.2
	100	21.3	0.001	13.6

^1^ *P* value was calculated using the log-rank test by comparing the survival rate of the untreated control group (0 mg/l phosphatidylcholine) to that of the phosphatidylcholine-treated group (10 or 100 mg/L phosphatidylcholine). ^2^% effects were calculated by (*C* − *p*)/*C* ^∗^100, where *p* is the mean lifespan of the phosphatidylcholine-treated group and *C* is the mean lifespan of the untreated control group. PC: phosphatidylcholine.

**Table 2:** Effect of phosphatidylcholine on age-related decline of motility.

Age (day)	Phase	Control (%)	10 mg/l PC (%)	100 mg/l PC (%)
5	1	100	100	100
	2	0	0	0
	3	0	0	0
	Dead	0	0	0
10	1	90.1 ± 3.33	95.6 ± 1.41	94.7 ± 3.53
	2	5.0 ± 1.64	3.7 ± 2.03	4.0 ± 2.31
	3	2.0 ± 1.10	0	0
	Dead	2.7 ± 1.80	0.7 ± 0.72	1.3 ± 1.33
15	1	51.2 ± 10.32	72.8 ± 22.29	62.3 ± 18.23
	2	11.7 ± 4.47	14.2 ± 4.79	19.2 ± 9.88
	3	17.4 ± 2.27	8.1 ± 5.00	7.9 ± 4.90
	Dead	19.7 ± 5.13	4.9 ± 2.79	10.6 ± 3.56
20	1	7.5 ± 1.13	24.0 ± 3.46^∗^	22.3 ± 4.91^∗^
	2	6.1 ± 0.55	23.3 ± 3.84^∗^	20.6 ± 3.71^∗^
	3	9.1 ± 1.15	11.7 ± 1.76	14.1 ± 2.88
	Dead	77.3 ± 2.60	41.0 ± 3.21^∗^	43.0 ± 5.25^∗^
25	1	0.67 ± 0.67	7.3 ± 1.86^∗^	3.7 ± 0.30^∗^
	2	0.33 ± 0.33	4.3 ± 2.60	7.2 ± 1.84^∗^
	3	5.29 ± 2.09	11.0 ± 3.79	8.1 ± 1.47
	Dead	93.7 ± 3.02	77.3 ± 2.03^∗^	81.0 ± 1.11^∗^

PC: phosphatidylcholine; ^∗^statistically significant compared to control (*P* < 0.05).

**Table 3:** Effect of phosphatidylcholine on A*β*-induced toxicity in *C. elegans.*

	PC (mg/l)	Time when 50% of worms were paralyzed (h)	*P* value^1^	% effect^2^
1st experiment	0	4.1		
	10	6.3	<0.001	55.7
	100	7.0	<0.001	71.3
2nd experiment	0	4.1		
	10	7.1	<0.001	71.8
	100	7.4	<0.001	77.8
3rd experiment	0	3.3		
	10	6.7	<0.001	106.1
	100	8.4	<0.001	157.7

^1^ *P* value was calculated using the log-rank test by comparing the rate of paralysis in the untreated control group (0 mg/l phosphatidylcholine) to that in the phosphatidylcholine-treated group (10 or 100 mg/l phosphatidylcholine). ^2^% effects were calculated by (*C* − *p*)/*C* ^∗^100, where *p* is the time when 50% of worms were paralyzed in the phosphatidylcholine-treated group and *C* is the time when 50% of worms were paralyzed in the untreated control group. PC: phosphatidylcholine.

**Table 4:** Effect of phosphatidylcholine on lifespan of wild-type N2 and long-lived mutants.

		Mean lifespan (day)		*P* value^1^	% effect^2^
		Control	100 mg/l PC		
N2	1st experiment	17.5	20.6	<0.001	17.6
	2nd experiment	21.6	24.9	<0.001	15.4
*age-1* (*hx546*)	1st experiment	29.2	28.9	0.970	-1.0
	2nd experiment	33.8	30.4	0.090	-10.1
*clk-1* (*e2519*)	1st experiment	22.2	25.4	0.013	14.7
	2nd experiment	22.0	25.8	<0.001	17.2
*eat-2* (*ad465*)	1st experiment	21.9	25.8	0.001	18.3
	2nd experiment	23.7	27.4	0.002	15.6

^1^ *P* value was calculated using the log-rank test by comparing the survival of the untreated control group to that of the phosphatidylcholine-treated group. ^2^% effects were calculated by (*C* − *p*)/*C* ^∗^100, where *p* is the mean lifespan of the phosphatidylcholine-treated group and *C* is the mean lifespan of the untreated control group. PC: phosphatidylcholine.

**Table 5:** Effect of *daf-16* knockdown on lifespan extension with phosphatidylcholine.

	RNAi	Mean lifespan (day)		*P* value^1^
		Control	100 mg/l PC	
1st experiment	EV	17.9	21.1	<0.001
	*daf-16*	15.5	15.6	0.622
2nd experiment	EV	18.5	20.2	0.005
	*daf-16*	15.7	16.4	0.255

^1^ *P* value was calculated using the log-rank test by comparing the survival rate of the untreated control group to that of the phosphatidylcholine-treated group. PC: phosphatidylcholine; EV: empty vector.

## Data Availability

The data used to support the results of this study are included within the article (and its supplementary materials). Requests for material should be made to the corresponding author.

## Supplementary Materials

Supplementary Materials Supplementary Figures: (1) effect of phosphatidylcholine on lifespan measured with dead bacteria; (2) effect of phosphatidylcholine on reproduction of *age-1*.

## References

[1] Pignolo R. J. (2019). Exceptional human longevity. *Mayo Clinic Proceedings*.

[2] Harman D. (1956). Aging: a theory based on free radical and radiation chemistry. *Journal of Gerontology*.

[3] Beckman K. B., Ames B. N. (1998). The free radical theory of aging matures. *Physiological Reviews*.

[4] Harper M. E., Bevilacqua L., Hagopian K., Weindruch R., Ramsey J. J. (2004). Ageing, oxidative stress, and mitochondrial uncoupling. *Acta Physiologica Scandinavica*.

[5] Shigenaga M. K., Hagen T. M., Ames B. N. (1994). Oxidative damage and mitochondrial decay in aging. *Proceedings of the National Academy of Sciences of the United States of America*.

[6] Fossel M. (1998). Telomerase and the aging cell: implications for human health. *Journal of the American Medical Association*.

[7] Shamanna R. A., Croteau D. L., Lee J. H., Bohr V. A. (2017). Recent advances in understanding Werner syndrome. *F1000 Research*.

[8] Sohal R. S., Agarwal A., Agarwal S., Orr W. C. (1995). Simultaneous overexpression of copper- and zinc-containing superoxide dismutase and catalase retards age-related oxidative damage and increases metabolic potential in *Drosophila melanogaster*. *The Journal of Biological Chemistry*.

[9] Pérez V. I., Bokov A., Remmen H. V. (2009). Is the oxidative stress theory of aging dead?. *Biochimica et Biophysica Acta (BBA) - General Subjects*.

[10] Jang M., Cai L., Udeani G. O. (1997). Cancer chemopreventive activity of resveratrol, a natural product derived from grapes. *Science*.

[11] Howitz K. T., Bitterman K. J., Cohen H. Y. (2003). Small molecule activators of sirtuins extend *Saccharomyces cerevisiae* lifespan. *Nature*.

[12] Gruber J., Tang S. Y., Halliwell B. (2007). Evidence for a trade-off between survival and fitness caused by resveratrol treatment of *Caenorhabditis elegans*. *Annals of the New York Academy of Sciences*.

[13] Long J., Gao H., Sun L., Liu J., Zhao-Wilson X. (2009). Grape extract protects mitochondria from oxidative damage and improves locomotor dysfunction and extends lifespan in a Drosophila Parkinson’s disease model. *Rejuvenation Research*.

[14] Marzetti E., Calvani R., Bernabei R., Leeuwenburgh C. (2012). Apoptosis in skeletal myocytes: a potential target for interventions against sarcopenia and physical frailty - a mini-review. *Gerontology*.

[15] Oh S. I., Park S. K. (2017). N-Acetyl-L-cysteine mimics the effect of dietary restriction on lifespan and reduces amyloid beta-induced toxicity in *Caenorhabditis elegans*. *Food Science and Biotechnology*.

[16] Kim S.-H., Kim B.-K., Park S.-K. (2018). Selenocysteine mimics the effect of dietary restriction on lifespan via SKN-1 and retards age-associated pathophysiological changes in Caenorhabditis elegans. *Molecular Medicine Reports*.

[17] Park J. K., Kim C. K., Gong S. K., Yu A. R., Lee M. Y., Park S. K. (2014). Acanthopanax sessiliflorus stem confers increased resistance to environmental stresses and lifespan extension in Caenorhabditis elegans. *Nutrition Research and Practice*.

[18] Won S. M., Cha H. U., Yi S. S., Kim S. J., Park S. K. (2016). *Tenebrio molitor* extracts modulate the response to environmental stressors and extend lifespan in *Caenorhabditis elegans*. *Journal of Medicinal Food*.

[19] van der Veen J. N., Kennelly J. P., Wan S., Vance J. E., Vance D. E., Jacobs R. L. (2017). The critical role of phosphatidylcholine and phosphatidylethanolamine metabolism in health and disease. *Biochimica et Biophysica Acta (BBA) - Biomembranes*.

[20] Kim M., Nevado-Holgado A., Whiley L. (2017). Association between plasma ceramides and phosphatidylcholines and hippocampal brain volume in late onset Alzheimer's disease. *Journal of Alzheimer’s Disease*.

[21] Zhou M. M., Xue Y., Sun S. H. (2016). Effects of different fatty acids composition of phosphatidylcholine on brain function of dementia mice induced by scopolamine. *Lipids in Health and Disease*.

[22] Yamanaka Y., Yoshida-Yamamoto S., Doi H. (2012). Microtubule formation and activities of antioxidative enzymes in PC12 cells exposed to phosphatidylcholine hydroperoxides. *International Journal of Molecular Sciences*.

[23] Morganti P., Guevara, Palombo (2012). A phosphatidylcholine hyaluronic acid chitin–nanofibrils complex for a fast skin remodeling and a rejuvenating look. *Clinical, Cosmetic and Investigational Dermatology*.

[24] Peto R., Peto J. (1972). Asymptotically efficient rank invariant test procedures. *Journal of the Royal Statistical Society. Series A (General)*.

[25] Kamath R. S., Fraser A. G., Dong Y. (2003). Systematic functional analysis of the Caenorhabditis elegans genome using RNAi. *Nature*.

[26] Johnson T. E. (1990). Increased life-span of age-1 mutants in Caenorhabditis elegans and lower Gompertz rate of aging. *Science*.

[27] Gomes M. J., Martinez P. F., Pagan L. U. (2017). Skeletal muscle aging: influence of oxidative stress and physical exercise. *Oncotarget*.

[28] Murphy C. T. (2006). The search for DAF-16/FOXO transcriptional targets: approaches and discoveries. *Experimental Gerontology*.

[29] Rea S. L., Wu D., Cypser J. R., Vaupel J. W., Johnson T. E. (2005). A stress-sensitive reporter predicts longevity in isogenic populations of Caenorhabditis elegans. *Nature Genetics*.

[30] Sanchez-Blanco A., Kim S. K. (2011). Variable pathogenicity determines individual lifespan in *Caenorhabditis elegans*. *PLoS Genetics*.

[31] Link C. D. (2006). *C. elegans* models of age-associated neurodegenerative disease: lessons from transgenic worm models of Alzheimer’s disease. *Experimental Gerontology*.

[32] Cohen E., Dillin A. (2008). The insulin paradox: aging, proteotoxicity and neurodegeneration. *Nature Reviews Neuroscience*.

[33] Wong A., Boutis P., Hekimi S. (1995). Mutations in the clk-1 gene of Caenorhabditis elegans affect developmental and behavioral timing. *Genetics*.

[34] Lakowski B., Hekimi S. (1998). The genetics of caloric restriction in Caenorhabditis elegans. *Proceedings of the National Academy of Sciences of the United States of America*.

[35] Wood J. G., Rogina B., Lavu S. (2004). Sirtuin activators mimic caloric restriction and delay ageing in metazoans. *Nature*.

[36] Ogawa T., Kodera Y., Hirata D., Blackwell T. K., Mizunuma M. (2016). Natural thioallyl compounds increase oxidative stress resistance and lifespan in *Caenorhabditis elegans* by modulating SKN-1/Nrf. *Scientific Reports*.

[37] Pathath A. W. (2017). Theories of aging. *International Journal of Indian Psychology*.

[38] Martins W. K., Gomide A. B., Costa É. T. (2017). Membrane damage by betulinic acid provides insights into cellular aging. *Biochimica et Biophysica Acta (BBA) - General Subjects*.

[39] Kirkwood T. B. L. (1977). Evolution of ageing. *Nature*.

[40] del Campo A., Jaimovich E., Tevy M. F. (2016). Mitochondria in the aging muscles of flies and mice: new perspectives for old characters. *Oxidative Medicine and Cellular Longevity*.

[41] Muller F. L., Song W., Liu Y. (2006). Absence of CuZn superoxide dismutase leads to elevated oxidative stress and acceleration of age-dependent skeletal muscle atrophy. *Free Radical Biology and Medicine*.

[42] Kumar J., Park K. C., Awasthi A., Prasad B. (2015). Silymarin extends lifespan and reduces proteotoxicity in C. elegans Alzheimer’s model. *CNS & Neurological Disorders - Drug Targets*.

[43] Hartwig K., Heidler T., Moch J., Daniel H., Wenzel U. (2009). Feeding a ROS-generator to Caenorhabditis elegans leads to increased expression of small heat shock protein HSP-16.2 and hormesis. *Genes & Nutrition*.

[44] Zhang X., Fu Z., Meng L., He M., Zhang Z. (2018). The early events that initiate *β*-amyloid aggregation in Alzheimer’s disease. *Frontiers in Aging Neuroscience*.

[45] Rasmussen J., Mahler J., Beschorner N. (2017). Amyloid polymorphisms constitute distinct clouds of conformational variants in different etiological subtypes of Alzheimer’s disease. *Proceedings of the National Academy of Sciences of the United States of America*.

[46] Friedman D. B., Johnson T. E. (1988). Three mutants that extend both mean and maximum life span of the nematode, Caenorhabditis elegans, define the age-1 gene. *Journal of Gerontology*.

[47] Kenyon C., Chang J., Gensch E., Rudner A., Tabtiang R. (1993). A C. elegans mutant that lives twice as long as wild type. *Nature*.

[48] Tatar M., Bartke A., Antebi A. (2003). The endocrine regulation of aging by insulin-like signals. *Science*.

[49] Dillin A., Hsu A. L., Arantes-Oliveira N. (2002). Rates of behavior and aging specified by mitochondrial function during development. *Science*.

[50] Colman R. J., Anderson R. M., Johnson S. C. (2009). Caloric restriction delays disease onset and mortality in rhesus monkeys. *Science*.

[51] Mair W., Dillin A. (2008). Aging and survival: the genetics of life span extension by dietary restriction. *Annual Review of Biochemistry*.

