# Supplementary material for: Phosphatidylcholine Extends Lifespan via DAF-16 and Reduces Amyloid-Beta-Induced Toxicity in Caenorhabditis elegans
Source: Oxid Med Cell Longev. 2019 Jul 11;2019:2860642. doi: 10.1155/2019/2860642 (PMC6657616; doi:10.1155/2019/2860642)
Supplement: Supplementary Materials — Supplementary Figures: (1) effect of phosphatidylcholine on lifespan measured with dead bacteria; (2) effect of phosphatidylcholine on reproduction of age-1. [file 2860642.f1.pdf]

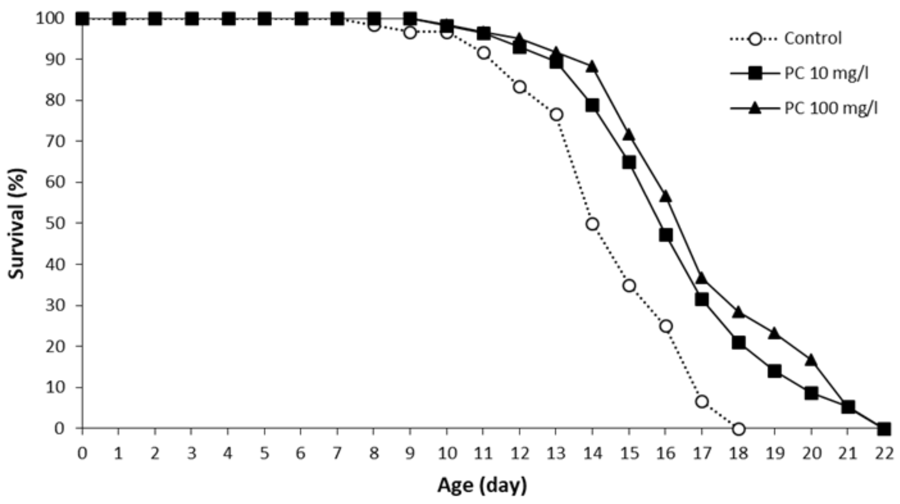

FIGURE S1: Effect of phosphatidylcholine on lifespan measured with dead bacteria. Lifespan was compared between the untreated control and worms treated with of phosphatidylcholine. All worms were grown on NGM plates containing dead bacteria with ampicillin (100 μg/ml). Lifespan was significantly increased by supplementation with both concentrations of phosphatidylcholine ($P < 0.05$). The log-rank test was employed for statistical analysis. PC, phosphatidylcholine.

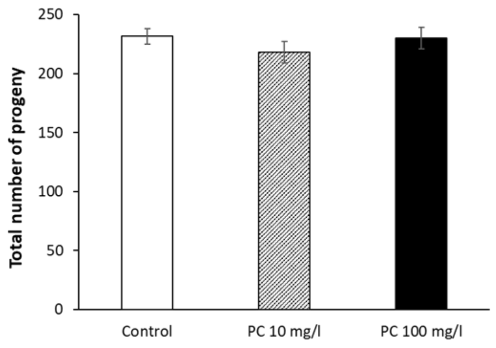

(a)

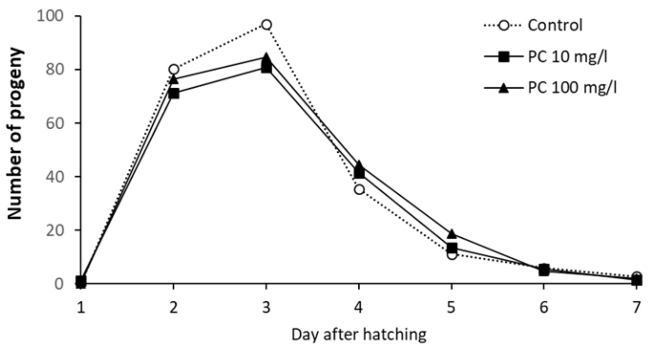

(b)

FIGURE S2: Effect of phosphatidylcholine on reproduction of *age-1*. (a) Total number of progeny produced during a gravid period was compared between the untreated control and phosphatidylcholine-treated groups. (b) Time-course distribution of progeny produced during a gravid period. Number of progeny was recorded every day until there was no progeny produced. Data indicate mean of 10 individual worms. Error bar indicates standard error. PC, phosphatidylcholine.
